# Lugol’s solution for preoperative management of a TSH/GH-secreting pituitary adenoma with suboptimal response to octreotide: a case report

**DOI:** 10.3389/fendo.2025.1698948

**Published:** 2026-01-15

**Authors:** Guiliang Peng, Xiaotian Lei, Weiling Leng, Feng Wu, Laiping Xie, Min Long, Liu Chen

**Affiliations:** 1 Department of Endocrinology, The First Affiliated Hospital (Southwest Hospital) of Army Medical University, Chongqing, China; 2 Department of Pathology, The First Affiliated Hospital (Southwest Hospital) of Army Medical University, Chongqing, China; 3 The Department of Nuclear Medicine, The First Affiliated Hospital (Southwest Hospital) of Army Medical University, Chongqing, China

**Keywords:** acromegaly, central hyperthyroidism, Lugol’s solution, octreotide, plurihormonal pituitary adenoma, somatostatin analogs, TSH-secreting adenoma

## Abstract

**Background:**

Thyroid-stimulating hormone pituitary adenomas (TSHomas) are a rare cause of central hyperthyroidism, characterized by abnormally high TSH levels, and typically respond to somatostatin analogue (SSA). We report a young patient with SSA-insensitive TSHoma where Lugol’s solution facilitated surgical preparation.

**Case presentation:**

A 28-year-old male patient presented with a 1.5-year history of headache and visual loss. Thyroid function revealed elevated levels of free triiodothyronine (FT3) 45.87 pmol/L, free thyroxine (FT4) exceeding 100 pmol/L, and non-suppressed TSH 6.66 mIU/L. Magnetic resonance imaging (MRI) suggested a large pituitary adenoma (19 × 25 × 23 mm). Initial long-acting octreotide treatment was ineffective in controlling hyperthyroidism and was discontinued after 5 months. Approximately 1 year after the initial presentation, reassessment showed persistently elevated thyroid hormone levels. A TSH suppression test indicated octreotide sensitivity at 55%. An oral glucose tolerance test (OGTT) suggested concomitant growth hormone (GH) excess. Preoperatively, treatment with short-acting octreotide, methimazole, and Lugol’s solution effectively controlled thyroid hormone levels. The patient subsequently underwent transnasal adenomectomy. Histopathology confirmed a PIT-1-positive pituitary adenoma, with TSH, GH, and prolactin (PRL) positivity. At the 3-month follow-up, thyroid hormone, GH, and insulin-like growth factor-1 (IGF-1) levels had normalized.

**Conclusions:**

This case highlights Lugol’s solution as a rescue therapy for SSA-insensitive TSH/GH co-secreting pituitary adenomas. Despite SSTR2/5 positivity, suboptimal response to octreotide suggests tumor heterogeneity or downstream signaling defects. Preoperative Lugol’s solution should be considered when SSAs and methimazole fail.

## Introduction

Thyroid-stimulating hormone pituitary adenomas (TSHomas) are a rare form of hyperthyroidism, typically presenting in individuals in the 40 to 60 age range (1). Diagnosis is often delayed due to inappropriate TSH elevation, leading to macroadenoma-related complications such as visual field defects. TSHomas express somatostatin receptors (SSTRs), particularly SSTR2 and SSTR5 (2), thereby rendering somatostatin analogue (SSA) therapy a primary treatment option. TSH normalization is achieved in approximately 90% of patients with TSH-secreting adenomas who are responsive to initial first-generation SSA therapies (3). Nevertheless, SSA insensitivity is exceptionally uncommon. Here, we present a case of a PIT-1-positive plurihormonal [TSH/growth hormone (GH)/prolactin (PRL)] adenoma leading to hyperthyroidism, where we outline the diagnostic challenges and successful preoperative biochemical control using Lugol’s solution combined with methimazole and octreotide. This management approach allows for clinical management refinement for SSA-insensitive TSH-secreting adenomas.

## Case presentation

In June 2023, a 28-year-old man presented to the First Affiliated Hospital of Army Medical University with a 1.5-year history of headaches and visual decline. He reported concurrent heat intolerance, excessive sweating, hand tremors, and unintentional weight loss symptoms (approximately 5 kg). He reported significant alcohol intake, consuming approximately 1,000 mL/day for 5 days weekly over 10 years. There was no family history of inherited diseases, including pituitary or thyroid disorders.

During examination, the patient’s temperature was 36.3 °C, pulse rate was 128 beats per minute, respiration rate was 20 beats per minute, blood pressure was 114/80 mmHg, and body mass index was 18.62 kg/m^2^. His eyes were slightly protruded but centrally positioned, with normal activity. The right eye appeared normal, and the cornea was transparent. The pupils were equal in size and round, with Modius’ sign observed in the left eye but von Graefe’s, Stellwag’s, and Joffroy’s signs were absent. Bilateral thyroid gland enlargement was grade I and his trachea was centered. No tremor or vascular murmur was palpable, and no acromegaly symptoms were observed.

### Hormone levels and imaging findings

Thyroid function demonstrated markedly elevated free triiodothyronine (FT3) 45.87 pmol/L, free thyroxine (FT4) exceeding 100 pmol/L, and TSH 6.66 mIU/L (**Table 1**). Thyroglobulin antibody (TGAb), thyroglobulin-microsome antibody (TMAb), thyroid peroxidase antibody (TPOAb), and thyrotropin receptor antibody (TRAb) were negative. GH level was 3.54 ng/mL, and insulin-like growth factor-1 (IGF-1) level was 302 ng/mL. Adrenocorticotropic hormone (ACTH), PRL, testosterone (T), luteinizing hormone (LH), and follicle-stimulating hormone (FSH) levels were within age-appropriate reference ranges. TRH stimulation testing was not performed due to reagent unavailability in China. No mutations were detected in the TSH receptor, thyroid hormone receptor-β, *AIP*, *MEN1*, *GPR101*, or *X-LAG* genes.

**Table 1:** Timeline showing thyroid function data in the patient.

Time	TSH (mIU/L)	FT3 (pmol/L)	FT4 (pmol/L)	GH (ng/mL)	IGF-1 (ng/mL)
20 June 2022	6.66	45.87	>100	3.54	302
27 June 2022 (long-acting OCT 30 mg Mon)	3.12	8.41	43.25		
28 July 2022	4.46	29.61	84.22	7.49	318.9
8 February 2023	7.32	>50	>100	4.74	382.6
Hospitalization					
3 July 2023	7.76	>50	>100	3.06	381.9
4 July 2023 (short-acting OCT 0.1 mg, Q8h)	4.06	48.14	>100	2.29	378.8
10 July 2023 (MTZ 10 mg bid)	3.77	9.65	37.98		
15 July 2023 (MTZ 10 mg tid)	3.69	9.6	32.54		
20 July 2023 (LS 0.3 mL tid)	3.53	8.72	37.89		
23 July 2023	4.75	5.56	25.78		
25 July 2023 (surgery)	5.16	5.71	23.19		
26 July 2023	0.055	4	34.92	2.74	193.5
31 July 2023	0.005	2.96	20.61	0.45	75.15
Follow-up					
11 September 2023	0.142	2.83	10.95	0.35	161.7
1 December 2023	0.547	3.67	13.94	0.53	146.7
24 May 2024	0.142	2.83	10.95	2.27	150.9
24 December 2024	0.547	3.67	13.94	2.16	139.5

OCT, Octreotide; MMI, methimazole; LS, Lugol’s solution; hormone (reference range): FT3, free triiodothyronine (3.1–6.8 pmol/L); FT4, free thyroxine (12–22 pmol/L); GH, growth hormone (0–5 ng/mL); IGF-1, insulin-like growth factor-1 (78.7–226 ng/mL); TSH, thyroid-stimulating hormone (0.27–4.2 mIU/L).

**Figure 1:**
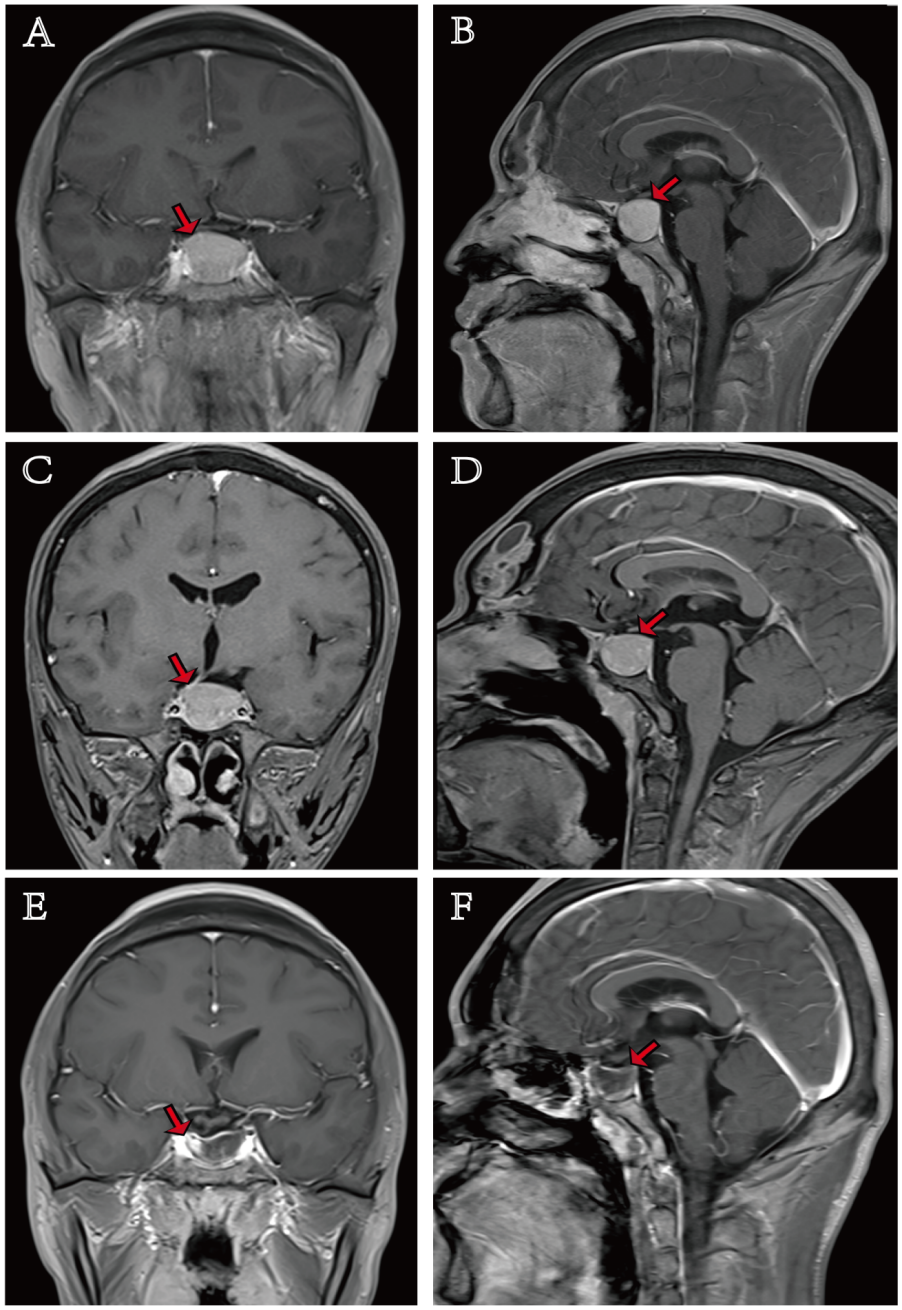
Serial contrast-enhanced pituitary magnetic resonance imaging (MRI). The initial MRI pituitary scan revealed a pituitary macroadenoma (19 × 25 × 23 mm). Initial diagnosis: **(A)** axial view and **(B)** sagittal view; 1 year after initial diagnosis: **(C)** axial view and **(D)** sagittal view; 3-month postoperative follow-up: **(E)** axial view and **(F)** sagittal view.

Contrast-enhanced cranial magnetic resonance imaging (MRI) identified a pituitary nodule (approximately 19 × 25 × 23 mm) exhibiting T1- and T2-isointense signal characteristics. The pituitary stalk was deviated to the right (**Figure 1**). Thyroid ultrasonography showed glandular enlargement with heterogeneous echotexture and scattered linear vascular signals. Several enlarged cervical lymph nodes were also noted. Thyroid scintigraphy revealed diffuse glandular enlargement with increased vascularity and markedly elevated 99mTc uptake. The uptake curve demonstrated a continuous rise over 1 min, consistent with hyperthyroidism.

### Diagnosis and treatment

Based on these findings, the initial diagnosis was a TSH-secreting pituitary adenoma. To control hyperthyroidism and mitigate perioperative thyroid storm risk, the patient was started on propranolol (20 mg three times daily) and short-acting octreotide (0.1 mg subcutaneously twice daily). Thyroid function tests repeated after 3 days showed substantial improvements: FT3 decreased to 8.41 pmol/L, FT4 to 43.25 pmol/L, and TSH to 3.12 mIU/L. Based on this initial favorable response, therapy was transitioned to long-acting octreotide (30 mg intramuscularly monthly).

Outpatient follow-up after 1 month revealed a biochemical deterioration with FT3 at 29.61 pmol/L, FT4 at 84.22 pmol/L, TSH at 4.46 mIU/L, and a GH level of 7.49 ng/mL. After regular long-acting octreotide use five times, the patient subsequently self-discontinued octreotide injections. At 6 months after the initial diagnosis, he experienced a significant biochemical relapse with FT3 exceeding 50 pmol/L, FT4 exceeding 100 pmol/L, TSH at 7.32 mIU/L, GH at 4.74 ng/mL, and IGF-1 at 382.6 ng/mL. Subsequently, the patient was lost to follow-up for a 6-month period.

Approximately 1 year after initial presentation, the patient requested a surgical intervention. Thyroid function tests revealed FT3 at >50 pmol/L, FT4 >100 pmol/L, and TSH at 7.76 mIU/L (**Table 1**). The GH level was 3.06 ng/mL (0–5 ng/L) and IGF-1 level was 381.9 ng/mL (78.7–226 ng/mL). ACTH was 37.86 pg/mL (5–60 pg/L), cortisol was 326.4 nmol/L (181.83–787.93 nmol/L), PRL was 4.36 ng/mL (2.64–13.13 ng/L), LH was 7.62 mIU/L (1.24–8.62 mIU/mL), FSH was 6.89 mIU/L (1.27–19.26 mIU/mL), and T was 6.72 ng/mL (1.75–7.81 nmol/L), all within normal limits. An octreotide suppression test (0.1 mg subcutaneously every 8 h) demonstrated 55% TSH inhibition at 24 h (decreased from 4.06 to 1.83 mIU/L) (**Table 2**). An oral glucose tolerance test (OGTT) revealed a basal GH level of 5.8 ng/mL, with a non-suppressed GH nadir of 3.22 ng/mL at 90 min, representing an inhibition rate of 38%. Repeat sellar MRI revealed a stable large pituitary nodule (19 × 25 × 21 mm) causing sellar expansion, erosion of the sellar floor, rightward pituitary stalk deviation, and indistinct margins with bilateral cavernous sinuses, and Hardy–Wilson classification was Grade 2B (**Figure 1**). Thyroid radioiodine uptake was elevated; 3H was 50% and 24H was 61.54%. Thyroid ultrasound and scintigraphy findings were unchanged.

**Table 2:** TSH suppression rate in short-acting octreotide suppression test.

Time (h)	TSH (mIU/L)	FT3 (pmol/L)	FT4 (pmol/L)	TSH suppression (%)
Baseline	4.06	58.14	>100	
2	3.52	47.34	>100	13.30
4	3.34	50.26	>100	17.73
6	3.12	49.67	>100	23.15
8	2.04	43.36	>100	49.75
16	3.49	38.93	>100	14.04
24	1.83	32.36	99.44	54.93

FT3, free triiodothyronine (3.1–6.8 pmol/L); FT4, free thyroxine (12–22 pmol/L); TSH, thyroid-stimulating hormone (0.27–4.2 mIU/L).

**Figure 2:**
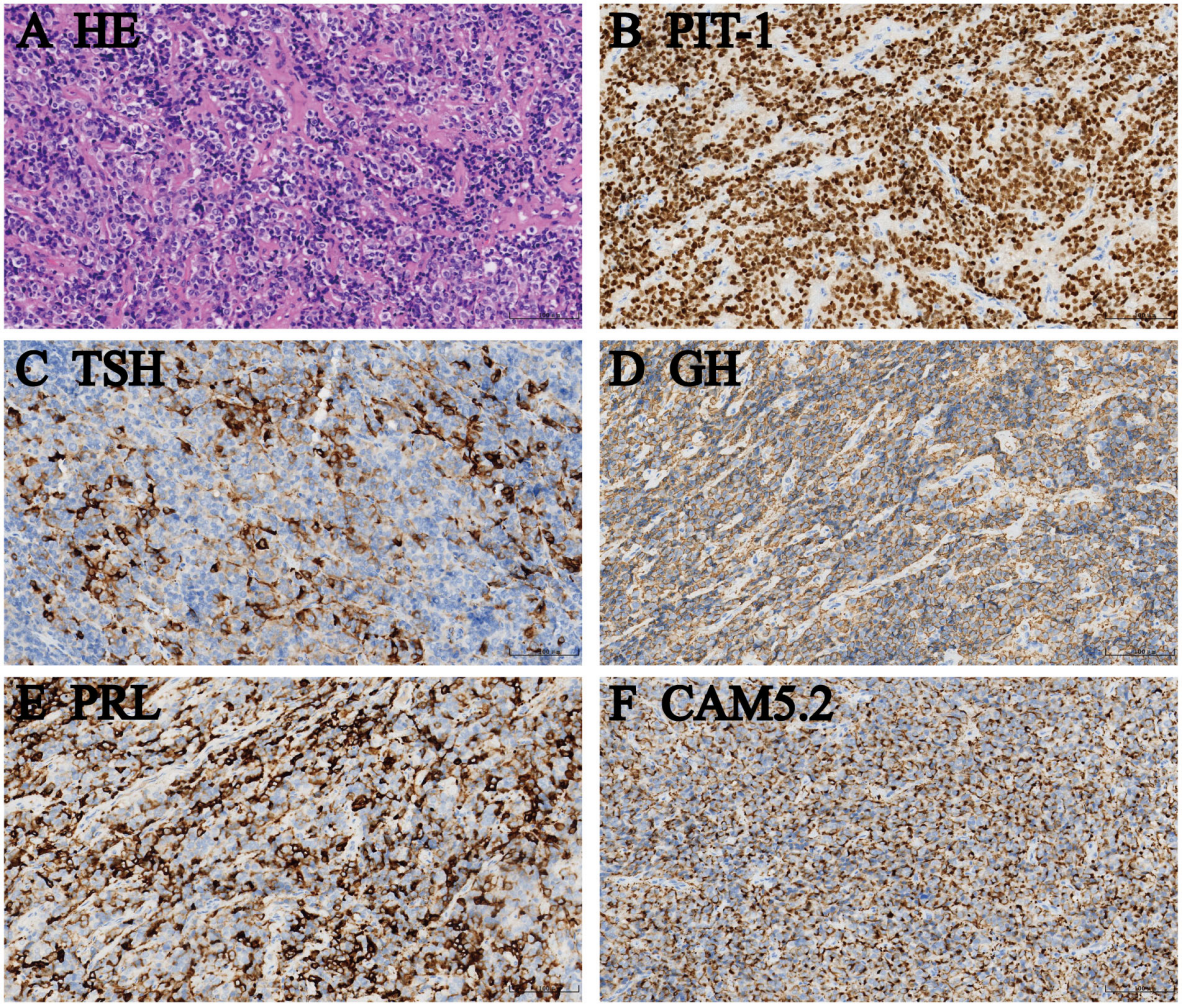
Immunohistochemical evaluation. **(A)** HE, **(B)** Pit-1, **(C)** TSH, **(D)** GH, **(E)** PRL, **(F)** CAM5.2, original magnification × 200.

Preoperative optimization commenced with resumed short-acting octreotide (0.1 mg subcutaneously every 8 h). After 7 days, thyroid function showed partial improvements with FT3 at 9.65 pmol/L, FT4 at 37.98 pmol/L, and TSH at 3.77 mIU/L. Methimazole was added to the regimen, starting at 10 mg twice daily and gradually increasing to 10 mg three times daily (30 mg/day in total). After 16 days on this combined therapy (octreotide and methimazole), further slight improvements were observed with FT3 at 8.72 pmol/L, FT4 at 37.89 pmol/L, and TSH at 3.53 mIU/L. Lugol’s solution [mixture containing 5% elemental iodine and 10% potassium iodide (0.3 mL three times daily)] was then initiated. After 20 days, thyroid hormones decreased, FT3 level was 5.56 pmol/L, FT4 level was 25.78 pmol/L, and TSH level was 4.75 mIU/L (**Table 1**). The patient then underwent a transsphenoidal tumor procedure. Histopathological examination confirmed a PIT-1-positive plurihormonal pituitary adenoma with TSH, GH, PRL, and LH immunopositivity (**Figure 2**). Immunohistochemical analysis revealed negativity for ACTH, SF-1, T-PIT, ERα, or FSH. Additionally, SSTR2 and SSTR5 staining showed strong positivity, while CAM 5.2 and p53 were also detected. The Ki-67 index was 1%. Postoperative follow-up at 1 week, 1 month, 3 months, and 12 months demonstrated sustained symptoms and biochemical remission with normalization of serum FT3, FT4, TSH, GH, and IGF-1 serum levels (**Table 1**).

## Discussion

TSHomas, first described in 1960, are rare endocrine tumors accounting for approximately 0.5%–3% of all pituitary tumors (1, 4, 5). TSH/GH co-secreting pituitary adenomas are an even rarer TSHoma sub-subtype, comprising 16.0%–19.7% of cases (3, 6). In our patient, typical hyperthyroid symptoms (e.g., palpitations) were accompanied by negative TGAb, TMAb, TPOAb, and TRAb, excluding Graves’ disease (GD) and Hashimoto’s thyroiditis. Additionally, TSH/GH co-secreting adenomas rarely coexist with GD, Hashimoto’s thyroiditis (7), and even thyroid carcinoma (8, 9). The detection of elevated GH levels (7.49 ng/mL) during initial long-acting octreotide therapy, inadequate GH suppression during OGTT (3.22 ng/mL at 2 h), and the definitive histopathological confirmation of GH co-secretion underscores the critical importance of actively screening for plurihormonality in patients diagnosed with TSHoma, even in the absence of overt acromegaly.

From the literature, the evidence supports a beneficial role for preoperative SSA therapy for normalizing hormone levels in hyperthyroidism associated with TSHomas (10–12). According to the 2022 WHO Classification of Pituitary Tumors (13), the PIT1-lineage pituitary neuroendocrine tumors (PitNETs) may show TSH, GH, and/or PRL positivity, thereby aligning with our histopathological findings. The presence of somatostatin receptors SSTR2 and SSTR5, primary targets of first-generation SSAs such as octreotide, as confirmed by our immunohistochemistry data, is consistent with the molecular profile expected for PIT-1 lineage PitNETs (14). However, the patient exhibited primary suboptimal responses to octreotide therapy, long-acting octreotide failed to maintain biochemical control upon initiation, and even short-acting octreotide achieved only partial biochemical control preoperatively, with a maximal TSH suppression rate of 55%. This suppression contrasts sharply with the greater than 80%–90% efficacy typically reported for SSA therapy in TSHomas (3, 15). This underscores a crucial clinical point: while SSTR expression is required for predicting SSA responses, this observation alone is insufficient to guarantee therapeutic efficacy. In TSH/GH co-secreting adenomas, octreotide was recently shown to suppress baseline GH and TSH levels by 79.1% and 94.7%, respectively. Notably, SSA treatment may induce TSH deficiency, though intervention is rarely required (16). Additionally, potential adverse effects are associated with SSA, and include gastrointestinal symptoms (diarrhea, nausea, and cholestasis), pruritus, bradycardia, and hyperglycemia (14, 17).

The successful implementation of preoperative multimodal medical therapy represents a significant aspect of this patient’s management. Achieving a euthyroid state is paramount for minimizing thyroid storm risk during pituitary surgery. This patient vividly illustrates the refractory nature of this particular tumor to standard first-line therapy, showing inadequate response not only to SSA monotherapy (long-acting and short-acting) but also to combined SSA and methimazole. Lugol’s solution (standardized preparation containing 5% elemental iodine and 10% potassium iodide) has been traditionally used for preoperative preparation in GD (18), where it rapidly suppresses thyroid hormone release via Wolff–Chaikoff effects. However, to the best of our knowledge, there is limited evidence of its therapeutic application in central hyperthyroidism secondary to TSH-secreting adenomas. In our patient who demonstrated suboptimal responses to octreotide-based therapy, Lugol’s solution was administered following multidisciplinary endocrine-tumor board discussion with the specific objective of rapidly normalizing free thyroid hormone levels and facilitate safe surgical intervention, despite the theoretical concern of potential TSH elevation due to iodine-induced feedback. Significant FT4 (from 37.89 to 23.19 pmol/L) and FT3 (8.72 to 5.71 pmol/L) reductions within 5 days of Lugol’s administration helped normalize TH levels. Intervening with Lugol’s solution intervention may be a valuable adjunctive therapeutic strategy in the preoperative management of patients with SSA-insensitive TSHomas requiring definitive surgery. Concomitantly, for PIT-1 lineage pituitary adenomas demonstrating suboptimal responses to first-generation SSAs like octreotide, pasireotide represents a rational alternative therapeutic option due to its broader SSTR affinity profiles, particularly its high binding affinity for SSTR5, which is frequently expressed in TSH/GH co-secreting adenomas (19). Unlike octreotide (primarily SSTR2-selective) (20, 21), pasireotide’s multi-receptor targeting (SSTR1, 2, 3, and 5) may overcome tumor heterogeneity and receptor downregulation that are commonly observed in plurihormonal tumors.

A comprehensive postoperative management strategy for PIT1-lineage PitNETs necessitates a stratified approach. For patients with residual disease following suboptimal resection or persistent biochemical abnormalities, continued SSA therapy remains a medical management cornerstone. However, given the PIT1-lineage PitNETs (i.e., TSH, GH, and PRL co-secretion), combination therapy with dopamine agonists, particularly cabergoline, which demonstrates activity at both D2 receptors and SSTR2/5, may provide synergistic hormone suppression (22). Furthermore, adjuvant radiotherapy is recommended for aggressive tumors with a Ki-67 index greater than 3% or cavernous sinus invasion to control tumor progression (23), though hormonal normalization may take several years to manifest. To monitor postoperative recurrence, the long-term monitoring of pituitary secreted hormones, such as TSH, GH, and PRL, and continuous MRI are performed. In young patients, such as our 28-year-old patient, genetic evaluation for potential underlying syndromes, including *MEN*, *AIP* mutations, or *X-LAG*, should be considered (24).

This case report had several limitations (1): octreotide sensitivity was not evaluated at initial admission (2); long-acting octreotide administration was not systematically monitored during follow-up; and (3) long-term postoperative remission awaits longer-term assessment.

In conclusion, we described a patient with central hyperthyroidism secondary to TSH/GH co-secreting adenomas. Suboptimal responses to first-line SSA therapy were shown by the patient. Initially, it was not controlled by long-acting octreotide, while at a subsequent readmission, an ineffective combination of short-acting octreotide (with a maximal 55% TSH suppression) and methimazole was administered. Biochemical management was eventually accomplished using Lugol’s solution prior to the operation. Histopathology confirmed a plurihormonal PitNET with immunopositivity for PIT1, TSH, GH, and PRL. Symptoms and biochemical remission were sustained at the 12-month postoperative follow-up.

## Data Availability

The original contributions presented in the study are included in the article/supplementary material. Further inquiries can be directed to the corresponding authors.
